# Transgender-specific developmental milestones and associated experiences of violence, discrimination, and stigma among Filipinx transgender women who are sexually active with men

**DOI:** 10.1371/journal.pone.0248248

**Published:** 2021-03-09

**Authors:** Arjee J. Restar, Aaron S. Breslow, Harry Jin, Ma Irene Quilantang, Olivia Sison, Amiel Nazer Bermudez, Maylin Palatino, Alexander Adia, Susan Cu-Uvin, Don Operario, Jennifer Nazareno

**Affiliations:** 1 Department of Behavioral and Social Sciences, Brown University School of Public Health, Providence, Rhode Island, United States of America; 2 The Philippine Health Initiative for Research, Service, and Training, Brown University School of Public Health, Providence, Rhode Island, United States of America; 3 Department of Epidemiology, Bloomberg School of Public Health, Johns Hopkins University, Baltimore, Maryland, United States of America; 4 PRIME Center for Health Equity, Albert Einstein College of Medicine, Bronx, New York, United States of America; 5 Health Equity Research Lab, Harvard Medical School, Cambridge, Massachusetts, United States of America; 6 Department of Behavioral Sciences, University of Philippines, Manila, Philippines; 7 College of Medicine, University of Philippines, Manila, Philippines; 8 College of Public Health, University of Philippines, Manila, Philippines; 9 Providence-Boston Center for AIDS Research, Providence, Rhode Island, United States of America; 10 Miriam Hospital, Department of Medicine, Providence, Rhode Island, United States of America; Hackensack Meridian Health, UNITED STATES

## Abstract

**Background:**

For transgender people, reaching transgender (trans)-specific developmental milestones, including recognizing and expressing one’s identity, plays an integral role in overall health, wellbeing, and the pursuit of gender affirmation. Yet trans people continue to face minority stressors, including structural violence (i.e., discrimination, violence, and stigma), which may interfere with the achievement of these milestones. Among trans women specifically, however, potential associations between gender developmental milestones and structural violence are not well characterized in the literature. In a sample of Filipinx (i.e., an inclusive term for describing non-binary genders in the Philippines) trans women who are sexually active with men (trans-WSM), we thus sought to: (a) describe the mean ages at which gender developmental milestones occur and (b) examine the associations between structural violence and mean ages at which at which Filipinx trans-WSM experience trans-specific developmental milestones.

**Methods:**

Using data from Project #ParaSaAtin, an online survey of Filipinx trans-WSM (n = 139), we mapped age-estimates per trans-specific milestones and then tested whether structural violence is associated with the mean age at which trans women experience trans-specific developmental milestones.

**Results:**

Overall, participants who reported higher levels of discrimination, stigma, and violence also experienced a later age for nearly each milestone (i.e., initial self-awareness of transfeminine identity, transfeminine expression in private, transfeminine expression in public, first consensual oral/vaginal/anal sex with a cisgender male partner, first consensual oral/vaginal/anal sex with a cisgender male partner as a trans women, and hormone integration) (all p-values <0.05). Of note, the single exception to this pattern was the non-significant association between stigma and initial disclosure of transfeminine identification to another person.

**Conclusion:**

Results are consistent with psychological literature outlining a temporal sequence of developmental milestones among young trans-WSM. For young trans-WSM in the Philippines, data from this study demonstrate significant associations between structural violence and the achievement of developmental milestones. These findings highlight the need for trauma-informed, strengths-based programming and institutional policies that measure and mitigate anti-trans violence.

## Introduction

The processes through which transgender (trans) women recognize and express their gender identity are not well characterized in the literature. However, a growing body of research demonstrates the importance of reaching trans-specific *developmental milestones* (e.g., disclosing one’s transfeminine identity to others; expressing one’s transfeminine identity in public, changing one’s name/gender marker on government documents) in shaping the trajectories of the health and overall wellbeing of trans persons [1–3]. Indeed, reaching such milestones may be key components of *gender affirmation*, or the process of taking social, medical, and/or legal steps to achieve internal and external recognition of one’s gender identity [4]. Among trans women, pursuing gender affirmation may be critical to mitigating the impact of gender minority stress, or the pernicious, biopsychosocial sequalae of contending with anti-trans stigma and discrimination [5]. Despite recent data suggesting the importance of meeting trans-specific developmental milestones for trans women [2], few studies have explored how achieving these important milestones may play a protective role against the adverse mental health impact of stigma and structural violence. Although our understanding is limited, there is a growing body of literature that characterizes a significant association between adverse mental health outcomes and experienced violence, as well as the negative impact of discrimination on educational attainment among transgender populations [6].

Similarly, a dearth of research has explored potential associations between structural violence and the achievement of developmental milestones key to gender affirmation. Such an inquiry may be critical, however, given emerging evidence that reaching developmental milestones is associated with positive mental health outcomes, particularly for trans women. In a recent study, trans persons in the United States who changed their gender markers on government documents (i.e., passport and state ID) to reflect their gender identity (i.e., pursued legal gender affirmation) reported lower levels of negative mental health outcomes such as serious psychological distress, suicidal ideation, and suicide planning compared to those who did not change their gender markers [3]. In contrast, some research has found that experiencing developmental milestones during adolescence may subject trans persons to minority stress in addition to the societal and peer pressures that are common during this stage of life [7]. A similar study found that trans persons who experienced milestones earlier in life (i.e., during adolescence) were less likely to attain a four-year degree compared to those who experienced milestones during childhood or adulthood [1]. Associations between developmental milestones and health/social vulnerabilities among trans adolescents may occur as a result of lack of family support or limited access to gender-affirming services for adolescents. These findings suggest the complex nature and impact of experiencing trans-specific developmental milestones on trans persons’ quality of life, necessitating further research to understand how to affirm trans persons and ameliorate trans-related stigma during the course of gender identity development.

A recent paper examined developmental milestones in a diverse sample of young trans women aged 16–29 in the United States (US) [2]. The developmental milestones explored in this paper included *internal processes* such as developing an initial awareness of one’s transfeminine identity and transfeminine expression in private, as well as *external or public processes* such as disclosure of transfeminine identity to others, transfeminine expression in public, having first consensual sex with a partner as a trans woman, and initiation of hormones. Findings revealed that, on average, the earliest milestone achieved was initial awareness of transfeminine identity at approximately 10 years of age. The remaining milestones were typically experienced during adolescence, between ages 12 to 18. Although the timing of internal milestones tended to occur at similar ages across racial/ethnic groups, trans women of color tended to experience external milestones earlier than White trans women. Authors suggested that whereas earlier external milestones can promote positive mental and behavioral health in the presence of supportive social contexts, earlier development may pose risks for young trans women who do not have robust support and/or social resources. Additionally, reaching these milestones may differ by context/region due to cultural and structural factors that might facilitate or inhibit trans affirmation. As such, the current paper aims to expand research beyond the United States to examine patterns of milestone development in other global settings.

Unclear in the literature is the potential exacerbating impact of structural violence, or discrimination, violence, and stigma, on the developmental trajectories of trans women. A key aspect of this inquiry is the potential role of structural violence, defined as “avoidable impairment of fundamental human needs” that stops individuals and/or groups from reaching their full potential [8]. Structural violence is one of many factors that perpetuates the marginalization of trans women and, consequently, may be associated with achievement of trans-specific developmental milestones [9]. In the US, some of the structural factors linked to adverse health outcomes among trans women include lack of nondiscriminatory policies across institutional domains (e.g., healthcare, schools, employment), lack of health care insurance coverage for transgender-specific services like hormone therapy, and lack of provider training and education [5, 10–13]. Similarly, a nascent body of literature has focused on trans women in the Philippines, where there is growing visibility and social recognition of this community as well as early findings documenting health and social vulnerabilities, including violence and discrimination among trans women [14–17]. For example, unique expressions of anti-trans structural violence among Filipinx (i.e., an inclusive term for describing non-binary genders in the Philippines) trans women were described in the context of healthcare settings, peer networks, educational settings, public retail establishments, as well as the Catholic Church. In a phenomenological study of Filipinx trans women [15], participants described being discriminated against and losing their jobs due to their feminine appearance, with employers citing company rules and policies on hair and dress codes. The instances described in these analyses demonstrate how structural violence and discriminatory policies not only discourages Filipinx trans women from exploring their gender identity and experiencing trans-specific developmental milestones, but also threatens the safety of those who do. While specific instances of structural violence have been described, there have not been any quantitative studies that describe how structural violence impacts trans women’s ability to experience important trans-specific developmental milestones in the Philippines or elsewhere.

The current study aims to fill gaps in knowledge about potential associations between structural violence and developmental milestones among trans women who are sexually active with men (trans-WSM) in the Philippines. Specifically, the study aims to explore such associations using data from a study with Filipinx trans-WSM. These analyses aimed to (a) describe the mean ages at which these trans-specific developmental milestones occur and (b) examine the associations between structural violence and mean ages at which at which Filipinx trans-WSM experience trans-specific developmental milestones. Identifying mean ages at which milestones occur holds potential to inform clinical outreach with young trans-WSM. Indeed, mapping age-estimates onto trans-specific milestone may reveal temporal intervention points to begin STI/HIV risk prevention, mitigate the impact of exposure to gender minority stress, and tailor programming to engender healthy development despite social vulnerabilities [18]. For Filipinx trans-WSM in particular, exploring how structural violence may interfere with health-promoting behaviors may hold implications for interventions to remove socioecological barriers across the lifespan.

## Methods

### Study design and participants

This analysis utilized data from a cross-sectional online survey, Project #ParaSaAtin, designed to characterize and examine access to HIV prevention and treatment services among Filipinx trans-WSM (n = 139). This analysis focused specifically on describing trans-specific developmental milestones and linkages to experiences of discrimination, stigma, and violence. The full study procedures have been described elsewhere [19]. Briefly, between June 2018 and May 2019, participants were recruited using venue-based sampling, study flyers, and snowball recruitment strategies via three local community-based organizations (CBOs) serving trans communities in the two highest HIV burden areas in the Philippines, which are Manila and Cebu metropolitan cities. Eligibility requirements included the following: (1) being 18 years old or above, (2) identify as a trans woman, (3) had condomless anal sex in the past year with a cisgender male partner, (4) currently living in Metro Manila or Cebu, and (5) demonstrated English and consent comprehension via a brief cognitive screening tool consisting of true/false questions about the consent form. All participants provided an electronic written informed consent and received a P300 ($5.58 USD) compensation for completing the survey. All of the study procedures were informed and deemed appropriate by the three CBOs involved as local study partners of this project. Study procedures were approved by the Brown University Human Research Protection Program Institutional Review Committee (IRB#: 1802001982). Electronic written informed consent was obtained from all individual participants included in the study.

### Measure items

Participants completed a one-time, 20-25-minute survey that included measures on socio-demographics, trans-specific developmental milestones and hormone history for gender affirmation, as well as experiences of discrimination, stigma, and violence. Due to cross-sectional nature of the survey, exposures and outcomes of interest were measured simultaneously, providing no temporal relationships between measures.

#### Sociodemographic

We surveyed sociodemographic information including: (1) age (18–24 years old, 25–29 years old, 30–34 years old, and 35 years old or more), (2) current living location (Metro Manila or Cebu), (3) current employment status (employed or unemployed), and (4) education (high school or below, some college, or college and beyond).

#### Trans-specific developmental milestones, and hormone history

We used Restar and colleagues’ [2] indicators of trans-specific developmental milestones. Specifically, we asked participants at what age did they first experience the following milestones: (1) self-awareness of their transfeminine identity, (2) disclosure of their transfeminine identity to others; (3–4) private and public expression of transfeminine identity; and (5–6) initial sexual experience (i.e., oral, vaginal, anal sex) with a cisgender man before and after transfeminine identification. To assess hormone history, we asked participants if they have ever taken feminizing hormones for gender affirmation. Among those who reported not ever taking feminizing hormones, we asked whether they have ever desired to use it in the past (yes/no), and reasons for why they have not started hormones. Among those had ever taken feminizing hormones in the past, we asked about the locations where they get it from (e.g., clinic, private doctor, on the street, online, from a friend, pharmacy).

#### Structural discrimination, stigma, and violence

As a proxy to measuring structural discrimination, we utilized the discrimination portion of Davidson’s (2016) Gender Inequality Scale [20], which is a 4-item true/false subscale that asks participants whether they have experienced losing their job, been denied health services, been denied work promotion, or removed from direct contacts with patients/workmates/classmates because of their LGBT identification (Cronbach α = 0.87). Scores were summed and dichotomized at the median (low discrimination: 0 ≤ 1.20, and high discrimination: 1.21 > 4). To measure structural violence, we adapted Woulfe and Goodman’s Violence and Identity Abuse Scale [21]. This is a 7-item scale (Cronbach α = 0.98) that asks participants whether they have experienced violent reactions from others such as: “Having had a person call them pejorative names that have to do with my LGBTQ status / Having had a person threatened to tell their employer, family, or others about their gender identity / Having had a person used their gender identity against them.” Scores were summed and dichotomized at the median (low stigma: 0 ≤ 15.03, and high stigma: 15.03 > 35). We utilized Hill and colleagues’ Transphobia, Homophobia and Genderism Scale [22] to assess structural stigma. This 15-item, 5-point Likert scale measures perceptions of social beliefs towards trans women (Cronbach α = 0.94). Participants were asked to rate from 1 = strongly disagree to 5 = strongly agree on scale items such as: “People believe that it is morally wrong for a cisgender male person to present herself as a woman in public / People around me have teased a transgender woman before of her masculine appearance or behavior.” Scores were summed and dichotomized at the median (low violence: 0 ≤ 60.57, and high violence: 60.57 > 76).

### Analysis strategy

We conducted descriptive analysis to describe patterns across our measures, and z-test procedures to identify mean age differences in trans-specific developmental milestones and hormone history by experiences of discrimination, stigma, and violence. We performed sensitivity analysis to determine the internal consistency of our scaled variables (i.e., discrimination, stigma, violence), providing Cronbach alphas, and dichotomized them at their median points. We utilized a two-tailed test (p<0.05) to determine statistical significance. All analyses were conducted using StataSE version 16.1 [23].

## Results

### Sample characteristics

Table 1 describes the sample sociodemographic characteristics and hormone history.

**Table 1:** Sociodemographic and hormone history(n = 139).

	All n (%)
**Demographics**	
Age (continuous, at the time of the survey)	
18–24	45 (33.58)
25–29	54 (40.30)
30–34	20 (14.93)
35+	15 (11.19)
Study Site	
Manila	110 (79.14)
Cebu	29 (20.86)
Currently Employed	
Yes	80 (57.55)
No	59 (42.45)
Education	
High School or below	55 (39.57)
Some College	23 (16.55)
College or beyond	61 (43.88)
**Hormones**	
Ever taken hormones for gender affirmation	
Yes	90 (64.75)
No	49 (35.25)
If Yes: which venues did you get hormones from^ (n = 90)	
A clinic / health center	27 (30.00)
A private doctor, private practice	12 (13.33)
On the street	7 (7.78)
Online	19 (21.11)
From a friend	90 (55.56)
Pharmacy	48 (53.33)
If No: Any desire to use hormones for gender affirmation in the past? (n = 49)	
Yes	17 (34.69)
No	32 (65.31)
If No: Reasons for not starting hormones for gender affirmation (n = 49)	
Not sure where to go to get hormones	21 (42.86)
I don’t have prescription for hormones	15 (30.61)
I can’t afford hormones	23 (46.94)

Note:

^ denotes check all that apply response option.

Most of the Filipinx trans-WSM in this study were between ages 25–29 years old (40.30%), reported currently residing in Manila (79.14%), were employed (57.55%), and had college-level education or beyond (43.88%).

The majority of the sample reported they had ever taken hormones for gender affirmation (64.75%). When asked about venues for hormone access, the most common sources of hormones included friends (55.56%), pharmacy (53.33%), a clinic or health center (30.00%), and online (21.11%). Few reported attaining their hormones from the streets (7.78%) or from a private practice (13.33%). Among those who reported they had never taken hormones (35.25%), about one-third (34.69%) reported desiring to use hormones for gender affirmation in the past. When asked about reasons for not starting hormones, common responses included not being able to afford them (46.94%), not sure where to go (42.86%), and not having a prescription (30.61%).

### Developmental milestones and experiences of discrimination, stigma, and violence

Table 2 describes the developmental milestones and experiences of discrimination, stigma, and violence.

**Table 2:** Trans-specific development milestones and experiences of discrimination, stigma, and violence (n = 139).

	All	Discrimination		Z statistic (p-value)	Violence		Z statistic (p-value)	Stigma		Z statistic (p-value)
		Low	High		Low	High		Low	High	
**Trans-specific development milestones, Mean ages (SD)**										
1. Initial self-awareness of transfeminine identity	13.50 (6.31)	13.16	14.20	**-5.62 (<0.001)**	13.05	13.83	**-4.42 (<0.001)**	13.28	13.71	**-2.47 (0.0135**)
2. Transfeminine identity disclosure to others	14.75 (5.28)	14.33	15.60	**-6.82 (<0.001)**	14.46	14.96	**-2.81 (0.0048)**	14.81	14.68	0.73 (0.4594)
3. Transfeminine expression in private	15.03 (5.99)	14.65	15.81	**-6.22 (<0.001)**	14.22	15.58	**-7.33 (<0.001)**	14.28	15.75	**-8.35 (<0.001)**
4. Transfeminine expression in public	16.77 (6.03)	16.50	17.32	**-4.39 (<0.001)**	16.45	17.01	**-3.14 (0.0016)**	16.25	17.28	**-5.91 (<0.001)**
5. First consensual oral/vaginal/anal sex with a cisgender male partner	15.15 (5.69)	14.82	15.87	**-5.60 (<0.001)**	14.81	15.41	**-3.44 (0.0006)**	14.74	15.55	**-4.66 (<0.001)**
6. First consensual oral/vaginal/anal sex with a cisgender male partner as a trans woman	16.11 (5.93)	15.61	17.16	**-8.27 (<0.001)**	15.41	16.64	**-6.96 (<0.001)**	15.49	16.69	**-6.85 (<0.001)**
7. Hormone integration (in age) (n = 90)	18.25 (3.86)	18.0	18.8	**-3.60 (<0.001)**	17.92	18.50	**-2.71 (0.0067)**	17.48	19.02	**-7.27 (<0.001)**

Note: outcomes dichotomized at median; SD = standard deviation.

On average, Filipinx trans-WSM in this sample reported initial awareness of their transfeminine identity at age 13.50 (SD = 6.31) years old. Filipinx trans-WSM reported initial disclosure of their transfeminine identification to another person within 1 year of their self-awareness (M = 14.75, SD = 5.28), and began private and public expression of their transfeminine identity within 2 to 4 years of their initial awareness (respectively, M = 15.03, SD = 5.99, and M = 16.77, SD = 6.03). The mean age for first consensual sex with a cisgender man was 15.15 (SD = 5.69) years old, and the mean age for first consensual sex as a trans woman with a cisgender man 16.11 (SD = 5.93) years old. Among those who reported ever taking hormones for gender affirmation (n = 90), hormone integration started on average by age 18.25 (SD = 3.86).

Mean age differences were observed for each developmental milestone by discrimination, stigma, and violence. Overall, participants who reported higher levels of discrimination, stigma, and violence also experienced a later age for nearly each milestone (i.e., initial self-awareness of transfeminine identity, transfeminine expression in private, transfeminine expression in public, first consensual oral/vaginal/anal sex with a cisgender male partner, first consensual oral/vaginal/anal sex with a cisgender male partner as a trans women, and hormone integration) (all p-values <0.05). Of note, the single exception to this pattern was the non-significant association between stigma and initial disclosure of transfeminine identification to another person.

## Discussion

This study describes the rans-specific developmental milestones in a community-recruited sample of trans-WSM in Manila and Cebu, Philippines. Our findings expand previous research concentrated in the US [1–3], to an international setting and sample of trans-WSM in the Asia-Pacific; to our knowledge, this study provides the first account of this topic in this region. We found that on average, these milestones are experienced largely during adolescence, which supports previous research on the need for psychological, social, and medical resources to support trans adolescents and highlights the need for gender-affirming interventions targeting when these milestones occur [1–3]. As reported in the previous study of US trans women [2], we found that internal milestones (e.g., self-awareness of transfeminine identity) occurred earlier than external milestones (e.g., disclosure of transfeminine identity to another person, public expression of their transfeminine identity). Notably, initial self-awareness in this Filipinx sample occurred on average at 13.5 years of age, older than the average 9.9 years of age for the US sample. The majority of milestones occurred between the ages of 14 to 17, including disclosure of transfeminine identity and initiation of sexual activity. Use of hormones occurred later, started on average at approximately age 18. It is important to note that these ages are reported as averages, and that previous research in other settings have documented some of these milestones such as initial self-awareness to occur as young as 3 years old for some transgender children [24].

Importantly. we also found that experiences of structural discrimination, stigma and violence were significantly related to the timing of these milestones. In particular, Filipinx trans-WSM who reported higher rates of structural discrimination, stigma and violence also reported meeting the majority of milestones significantly later in life, with the exception of initial disclosure of transfeminine identification to another person. These findings urge for further programmatic support for adolescents who might be undergoing changes and stress related to trans-related identity development. Local community services and programming should encourage healthy developmental milestones for young Filipinx trans-WSM and must center and prioritize the psychological impact of violence, stigma, and discrimination. Gender-affirming sexual health services and programs mitigating the adverse impact of structural violence are needed to support trans adolescents in the Philippines. Perhaps more critically, it is critical for national laws along with institutional-level policies to adopt anti-transphobia and protect transgender people from discrimination, stigma, and violence across social institutions like healthcare, schools, and employment/labor. Currently, while there are anti-discrimination ordinances for transgender people at the local level, transgender populations continue to face legal challenges in the Philippines at the national level, leaving this population vulnerable to acts of discrimination on the basis of gender identity and expression [25]. As such, along with gender-affirming community services and programming, policies and laws that prevent gender-based anti-discrimination could support trans people and prevent delays in their trans-related identity development.

Previous research has reported that adolescents in the Philippines tend to initiate sexual intercourse at later ages compared with adolescents in other countries in the Global South, which might be due to pervasive cultural and religious norms in the Philippines that discourage premarital sex [26]. However, participants in this study reported age of sexual debut at an average 15 years old, commensurate with average age of sexual debut in a recent study with US trans women [2]. The average of first consensual sex among Filipinx trans-WSM in the current sample is younger compared with the national average (17 years old) in the Phillipines. Of note, it is possible the *true* mean age of first sex in this sample may be lower than reported, as this survey inquired only about consensual sex. Our findings point to the need for future research to understand dynamics of sexual consent among young Filipinx trans-WSM and their sexual partners, as well as the need for gender-affirming sexual health education programs for this population.

Perhaps most notable, participants who reported high (compared to low) levels of violence and stigma also reported older ages at which developmental milestones were met. There are multiple offer possible explanations for this observed association, which warrants further examination in subsequent research. One possible explanation could be that facing these negative experiences may lead trans-WSM to delay the process of important external gender-related discoveries and attainment of these milestones, i.e., pursuing social, legal, and medical gender affirmation. For example, experiences of discrimination in health care settings, including provider attitudes towards transgender people, likely prevent trans-WSM from seeking and/or initiating integration of hormone intervention [27, 28], and exposure to social or familial reprimands for violating binary gender roles may lead to self-censorship with regard to gender identity exploration and development. Another possible explanation could be that trans-WSM who achieve gender identity milestones relatively early are likely to benefit from gender affirming resources that keep them relatively protected from structural discrimination, stigma, and violence, compared to those who undergo milestones later and do not gain access to gender-affirming resources [29]. Future longitudinal and qualitative work could delineate the directionality of these postulations, including the extent to which structural discrimination, stigma, and violence may inform these trans-specific developmental milestones, and vice versa. Lastly, it is notable that the only nonsignificant association in our findings is the association between stigma and transfeminine identity disclosure to others. Research on the psychological role of identity disclosure for trans people is mixed, with disclosure posing both positive rewards (i.e., receiving affirmation and support, building emotional connection) and potential adverse risks (i.e., increasing likelihood of interpersonal and structural violence) [30]. Disclosure has been described as a form of “stigma management” for some trans people, that is, some trans individuals who are visually conforming to societal norms of binary gender, often known as “passing privilege” or “living stealth,” may continue to choose to not disclose their gender identity to avoid stigma, violence, and discrimination [5, 31–33]; our results add further support to the idea that intentional identity disclosure may be a resilience strategy to manage the impact of structural violence and stigma for some Filipinx trans-WSM as well.

It is noteworthy that while there are some developmental milestones that are independent of each other—for instance, trans-WSM choosing to not integrate hormone therapy as part of their trans-specific developmental goals [34]–a portion of women in this sample did express desiring to start hormone integration but experienced barriers to initiation. Specifically, among those who reported not having a history of hormone use, about a third (34.69%) reported desiring to use them for gender affirmation. Indicated barriers for initiating hormones in this sample included lack of information for available resources, not having access to a provider for hormone prescription, and financial cost; these barriers corroborate previous research documenting barriers to hormone use among trans women in other global settings [35–39]. Our findings, therefore, suggest that addressing access to hormones among young Filipinx trans-WSM, particularly those who are from low-income background and not fully connected to care but desire to start their initiation, must include providing educational information, referrals to providers, and eliminating cost related to hormone medications and clinical visits.

### Limitations

This study has several limitations. First, the cross-sectional nature of the data did not allow us to establish temporality and directionality between our exposure and outcome variables. Second, the secondary data from a parent study that aimed to understand HIV-related behavioral risks of trans women who are sexually active with cisgender men, resulting in a limited sample that does not represent and omit a large proportion of trans women who have not yet experienced or have chosen to not engage in sexual activities with a cisgender male partner. Third, it is possible that trans-WSM in this sample may experience other gender beyond or besides their transfeminine identity at an earlier age, as such future constructions of measurements should further characterize these experiences and strive to situate or contextualize such experiences locally to illuminate the fluidity of gender in this population in the Philippines. Fifth, given our measurements were assessed at the individual-level to understand experiences related to structural factors, this study can be further strengthened by having multiple sources of data that are not reliant on self-report (e.g., evaluation of facility-level policies, analyzing institutional records on discrimination reports) to accurately examine structural factors. Lastly, as Filipinx trans-WSM represent a portion of a wide array of trans identities and gender diverse communities [40–42], future research is needed for a more comprehensive overview of trans-specific developmental milestones across the diverse range of trans identities in the Philippines.

## Conclusion

This study is among the first to characterize trends in key developmental milestones among a sample of young trans-WSM in the Philippines. This study maps differences in the ages at which Filipinx trans-WSM living in Manila and Cebu affirmed their gender both internally and externally. Results are consistent with psychological literature outlining a temporal sequence of development among young trans and cisgender girls and women, including recent US data suggesting young trans women may first meet private/internal developmental milestones (e.g., developing a congruent, internal transfeminine identity) followed by a later-in-life achievement of external milestones (e.g., disclosing one’s trans identity to others) [2]. The achievement of such milestones has been shown to be potentially protective for the mental health of trans women, with studies demonstrating that taking steps to pursue both internal and external gender affirmation plays a mitigating role in the association between minority stressors and adverse health outcomes [43]. Results from the current study build on these findings with trans-WSM in the Philippines, specifically by demonstrating a positive association between gender minority stress (i.e., experiencing high levels of structural violence) and a later-in-life pursuit of gender affirmation through the achievement of these important milestones.

Filipinx trans-WSM in this sample tend to report an early-in-adolescence awareness of their gender identities, followed by a developmental process throughout their tends that includes steps toward identity disclosure and expression, hormone use (when accessible and desired), and sexual behaviors. Unique to trans-WSM’s experiences—both in the Philippines and globally—is the burden of contending with and managing structural anti-trans violence. Young trans-WSM are tasked not only with surviving adolescence—itself a profoundly difficult developmental rite of passage—but also with fostering and sustaining a positive, resilient sense of self despite a hostile environment. Indeed, structural violence has been shown to paradoxically stunt *and* accelerate sexual, gender, socioemotional, and cognitive development among all children and young adults. For young trans-WSM in the Philippines, data from this study suggest a significant association between the level of structural violence Filipinx trans-WSM experience and the age at which they achieve gender-related milestones. This key finding does not necessarily suggest that trans-WSM who experience higher levels of structural violence do not achieve milestones. More precisely, these data reveal that participants who experienced higher levels of structural violence achieved developmental milestones at slightly older ages: approximately one year later than those with lower levels of experiencing structural violence. Because data are not longitudinal, results do not necessarily indicate that violence *caused* a delay in development. These findings do, however, highlight a need for trauma-informed, strengths-based programming and institutional policies that measure and mitigate anti-trans violence, which is associated in this study with an approximately one-year delay in development. Finally, this study calls for an expansion in knowledge about the needs and strengths of trans communities beyond the US: gaps that can only be filled through earnest engagement with local community leaders already engaged in the struggle for gender health equity and trans and gender diverse liberation [5, 30].
